# A dual PMMA/calcium sulfate carrier of vancomycin is more effective than PMMA‐vancomycin at inhibiting *Staphylococcus aureus* growth *in vitro*

**DOI:** 10.1002/2211-5463.12809

**Published:** 2020-03-11

**Authors:** Shanchao Luo, Tongmeng Jiang, Lina Long, Yingnian Yang, Xiaoping Yang, Lan Luo, Jinli Li, Zhiyu Chen, Chongqi Zou, Shixing Luo

**Affiliations:** ^1^ Yulin Orthopedics Hospital of Chinese and Western Medicine Yulin China; ^2^ Guangxi Postdoctoral Innovation Practice Base Beihai People’s Hospital Beihai China; ^3^ Postdoctoral Mobile Station of Clinical Medicine Guangxi Medical University Nanning China; ^4^ Department of Orthopaedics Affiliated Hospital of Guilin Medical University China

**Keywords:** antibacterial properties, antibiotic delivery system, antibiotic release, calcium sulfate, PMMA, vancomycin

## Abstract

Both antibiotic‐impregnated poly(methyl acrylate, methyl methacrylate) (PMMA) and antibiotic‐impregnated calcium sulfate have been successfully used as local antibiotic delivery vehicles for the management of chronic osteomyelitis. Here, we examined the antibiotic elution characteristics and antibacterial properties of a composite drug delivery system consisting of PMMA/calcium sulfate carrying vancomycin (dual carrier‐v) against *Staphylococcus aureus*, with PMMA loaded with vancomycin (PMMA‐v) as a control. Vancomycin gradually degraded from dual carrier‐v and PMMA‐v up to about 8 and 6 weeks, respectively. At different elution time points, the inhibition zones of the dual carrier‐v were larger than the inhibition zones of the PMMA‐v (*P *< 0.05). The colony inhibition rate of the dual carrier‐v was 95.57%, whereas it was 77.87% for PMMA‐v. Scanning electron microscopy was used to demonstrate biofilm formation on the surface of plates treated with vancomycin‐unloaded PMMA, whereas there was no biofilm formation on the surface of plates treated with dual carrier‐v or PMMA‐v. The dual carrier‐v was more effective at antibacterial adhesion at each time point after immersion in simulated body fluid as compared with PMMA‐v (*P* < 0.05). In conclusion, our results suggest that the dual carrier‐v can release higher concentrations of antibiotics and inhibit bacteria growth more effectively *in vitro* as compared with PMMA‐v. The dual carrier‐v thus may have potential as an alternative strategy for osteomyelitis management.

## Graphical abstract

A dual poly(methyl acrylate, methyl methacrylate) (PMMA)/calcium sulfate carrier of vancomycin is more effective than PMMA‐vancomycin at inhibiting *Staphylococcus aureus* growth *in vitro*. The PMMA/calcium sulfate carrying vancomycin thus may have potential as an alternative strategy for the management of osteomyelitis.

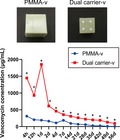

**Abbreviations**
ANOVA: analysis of variance
CFU: colony‐forming unit
dual carrier‐v: PMMA/calcium sulfate carrying vancomycin
M‐H: Mueller‐Hinton
NB: nutrient broth
PMMA: poly(methyl acrylate, methyl methacrylate)
PMMA‐n: vancomycin‐unloaded PMMA
PMMA‐v: PMMA loaded with vancomycin
SBF: simulated body fluid
SEM: scanning electron microscopy

Chronic osteomyelitis, especially resulting from multidrug‐resistant bacteria, is still a refractory disease and one of the major challenges for orthopedic surgeons, even though great progress has been made in surgical treatment and antibiotics for chronic osteomyelitis [1]. Local antibiotic‐loaded delivery systems are commonly adapted as effective vehicles for chronic osteomyelitis, to maintain antibiotics at a high level at the local site of osteomyelitis [2, 3]. Antibiotics‐loaded poly(methyl acrylate, methyl methacrylate) (PMMA) was the effective antibiotic carrier system to control chronic osteomyelitis, because it can provide the following advantages: a high releasing level of antibiotics at the local site of infection, synchronously eliminating the surgically debridement‐derived dead space, low toxicity and less in serum [2, 3, 4]. Although PMMA has been widely studied, some of its disadvantages have been discovered by many studies. The main drawback of antibiotics‐loaded PMMA is the superfluous drugs at the initial releasing stage, which thus contributed to a deficient concentration for therapeutics at the later stage [5, 6]. Due to the nonbiodegradable property of PMMA, the continuous reduction of antibiotics released by PMMA may result in proliferation of bacteria at the local sites and forming biofilms, which would be beneficial for the production of resistant bacteria and the recurrence of infection [7, 8].

Given the disadvantages of antibiotics‐loaded PMMA, antibiotics‐loaded calcium sulfate is one of the top attractive absorbable delivery systems used for chronic osteomyelitis [3, 9]. Similar to PMMA, calcium sulfate synchronously eliminates the surgically debridement‐derived dead space; in addition, it can completely release antibiotics even after complete degradation and does not provide matrix for the colonization of bacteria. Antibiotics‐loaded calcium sulfate can release antibiotics via zero‐order kinetics and layer‐by‐layer surface degradation. Thus, calcium sulfate provides a sustainable releasing rate of antibiotics and remains at a therapeutic dose for extended periods, which is not conducive to bacterial growth [10]. However, antibiotics‐loaded calcium sulfate also has intrinsic drawbacks, such as its deficient mechanical support for the bone structure, which is a critical factor for chronic osteomyelitis [11]. Due to the nondegradable characteristics, antibiotics‐loaded PMMA can provide immediate or sustained mechanical stabilization for the bone structure or is conducive to the stabilization maintenance of the bone structure.

Vancomycin is one of glycol‐peptide antibiotics that are anti‐gram‐positive bacteria and is especially suitable for fight against methicillin‐resistant *Staphylococcus aureus*, which is the major pathogen of osteomyelitis [12]. In our previous clinical study, the composite drug delivery system of PMMA/calcium sulfate carried vancomycin could gain more effectively clinical anti‐infection for chronic osteomyelitis [3]. However, further research is needed to evaluate the antibiotic release characteristics and the antibacterial properties of the composite drug delivery system. Thus, this work aimed to explore the antibiotic release characteristics and the antibacterial properties of the composite drug delivery system that consisted of PMMA/calcium sulfate carried vancomycin [termed here as PMMA/calcium sulfate carrying vancomycin (dual carrier‐v)] *in vitro* compared with PMMA loaded with vancomycin (PMMA‐v).

## Materials and methods

### Materials

Vancomycin (CAS No. 1404‐93‐9; molecular weight: 1486) was purchased from Sigma‐Aldrich (Shanghai, China). Stimulan® calcium sulfate powder (Stimulan® kit) was purchased from Biocomposites Ltd (Keele, Staffordshire, UK). PMMA cement (PALACOS® MV) was purchased from Heraeus Medical GmbH (Wehrheim, Germany). *S. aureus* subsp. *aureus* (*S. aureus*, CICC ®10301) was purchased from China Center of Industrial Culture Collection (Beijing, China).

### Preparation of drug delivery systems

**Fig. 1:**
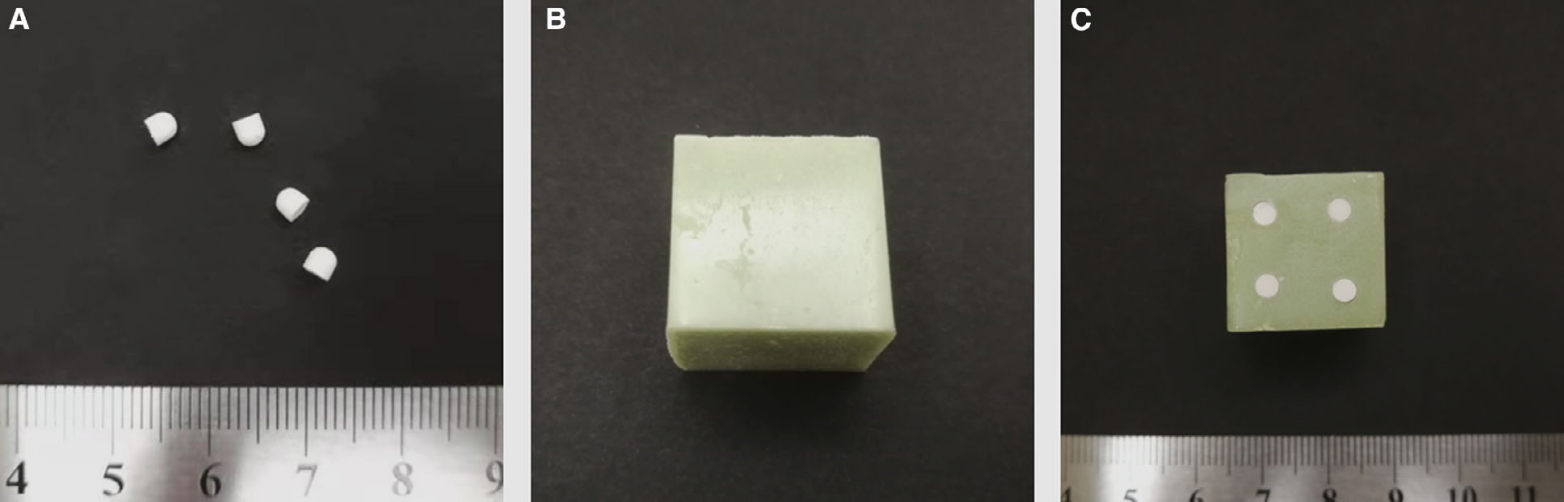
Different cargo carriers. (A) Vancomycin‐loaded calcium sulfate pellets. (B) Vancomycin‐loaded PMMA (PMMA‐v). (C) Dual carrier consisted of vancomycin‐loaded PMMA/calcium sulfate (dual carrier‐v).

Vancomycin‐loaded calcium sulfate pellets were obtained according to the following instructions. In short, 1000 mg vancomycin powders and 5 mL Stimulan® calcium sulfate powders were added into 3 mL sterile water. After thoroughly mixing for about 30 s, a smooth paste was formed. Then, a hemisphere at diameter of 3.0 mm was generated by putting the aforementioned paste into a mold for about 15 min. Finally, the mold was discarded to drop hemisphere after it cured completely (Fig. 1A).

PMMA‐v was obtained according to the following procedures under sterile conditions. One sachet of 40 g PALACOS® MV containing 38.3 g PMMA and 2000 mg of vancomycin powders was added into 20 mL of sterile liquid provided by the manufacturer. After mixing thoroughly, the mixed paste was then spread into a flexible rubber mold to make a cuboid element at a length of 15.0 mm, width of 15.0 mm and height of 12.0 mm. Finally, the mold was discarded to release elements after it cured completely (Fig. 1B). The PMMA element’s lack of vancomycin was also provided in the same way.

Four calcium sulfate cubes carried with vancomycin were then embedded into one PMMA element to obtain the dual carrier‐v, which was constructed of PMMA/calcium sulfate carried vancomycin (Fig. 1C).

### Preparation of simulated body fluid

The simulated body fluid (SBF) was prepared through NaHCO_3_ (0.355 g), NaCl (8.035 g); K_2_HP0_4_·3H_2_0 (0.231 g), MgCl_2_·6H_2_O (0.311 g), KCl (0.25 g), Tris (6.118 g), Na_2_SO_4_ (0.072 g), CaCl_2_ (0.292 g) and 1.0 m HCl (39 mL) dissolved in 1000 mL of distilled water (pH 7.4).

### Antibiotic elution *in vitro*

The standard drug curve was first established by high‐performance liquid chromatography–mass spectrometry (the detection limit was 3.125 μg·mL^−1^). The HPLC analyses were performed as described previously [13].

Samples of the dual carrier‐v or the PMMA‐v were immersed in sterile polyethylene containers containing 10 mL SBF (pH 7.4) at 37 °C, respectively. The SBF solution was semiquantitatively refreshed at the following different times: 4 h, 12 h, 24 h, 48 h, 96 h, 1 week, 2 weeks, 3 weeks, 4 weeks, 5 weeks, 6 weeks, 7 weeks and 8 weeks after samples immersed in SBF. The eluents were frozen at −4° C for further analysis. The vancomycin concentration in the sample eluents was determined through HPLC at the above different time points, according to the standard curve of vancomycin.

### Bacteria and growth conditions

The *S. aureus* was inoculated in the nutritious gravy agar (CICC No. 10301, Medium No. CM0002, China Center of Industrial Culture Collection; peptone 5.0 g, beef extract 3.0 g, NaCl 5.0 g, agar 15.0 g, distilled water 1000 mL, pH 7.0–7.2; http://english.china-cicc.org/goods.php?id=125546) at 37° C for 24 h and was retained for subsequent experiments after morphological identification as a pure culture. A single colony with good growth state was inoculated into the nutrient broth (NB) medium and cultured for 48 h under the same conditions, and the concentration of the suspension was adjusted to 1 × 10^3^, 1 × 10^5^ and 1 × 10^8^ colony‐forming units (CFUs) per milliliter by using the NB.

### Antibacterial properties by inhibition zone

The filter paper discs with a diameter of 6 mm were prepared and then sterilized with ethylene oxide. Five colonies were selected from blood agar plates incubated for 24 h. The bacterial suspension was prepared using the above colonies for inoculation. Then the bacterial concentration of the bacterial suspension was adjusted to 3 × 10^8^ CFUs·mL^−1^ (0.5 McFarland). The bacterial suspension was taken up with a sterile cotton swab, and the excess bacterial suspension in the cotton swab was squeezed on the wall of the culture tube. The cotton swab bacteria were coated on the surface of the entire Mueller‐Hinton (M‐H) agar plate, and the plate was rotated by 90 degrees each time to ensure uniform coating. The filter paper discs with a diameter of 6 mm were impregnated with 30 μL serial SBF eluents, which were semiquantitatively extracted from SBF at the following different times: 4 h, 12 h, 24 h, 48 h, 96 h, 1 week, 2 weeks, 3 weeks, 4 weeks, 5 weeks, 6 weeks, 7 weeks and 8 weeks after samples immersed in SBF. The vancomycin‐unloaded PMMA (PMMA‐n) was immersed in SBF, and the extraction was used as a negative control in antibacterial properties. The filter paper discs were placed on the earlier‐mentioned coated bacteria M‐H agar, and the Petri plates were incubated for 24 h at 37° C. The diameters of the inhibition zones around the filter paper discs were measured with vernier calipers.

### Antibacterial properties by colony inhibition

The samples of this colony inhibition experiment were divided into three groups: PMMA‐n, PMMA‐v and dual carrier‐v. The NB suspension was prepared with 3 × 10^8^ CFUs·mL^−1^ (0.5 McFarland) of the *S. aureus*. Then 100 μL of the NB suspension was placed on the surface of the different samples and covered with a cover glass to uniformly distribute the bacterial liquid, and was cultured in a constant temperature incubator at 37° C for 12 h. In the group of dual carrier‐v, the NB suspension was placed on the surface implanted with vancomycin‐loaded calcium sulfate pellets. The earlier‐mentioned samples with the NB bacterial suspension were respectively placed in the sterile NB liquid medium and then ultrasonically irradiated for 2 min at 40 kHz to elute the bacteria adhering to the surface of the samples. The bacterial suspension obtained after ultrasonically irradiating was diluted with sterilized physiological saline by a gradient dilution method. Then, 100 μL of the earlier‐diluted bacterial suspension was added to the Petri dish containing 10.0 mL of nutrient agar at 37.0 ± 1.0° C. The eluted bacteria suspension was effectively mixed with the nutrient agar by rotating the dish. After the nutrient agar concreting, the Petri dish was incubated for 24 h at 37° C and the CFUs were counted. The antibacterial rate was evaluated by the following formula: Antibacterial rate [14] = (CFUs in the control group − CFUs in the experiment group)/CFUs in the control group × 100%.

### Antibacterial adhesion evaluated by scanning electron microscopy

Scanning electron microscopy (SEM) (VEGA3LMU; TESCAN, a.s., Shanghai, China) was used to evaluate the impact of bacterial adhesion on the surface of the different drug delivery samples at an accelerating voltage of 4.0 kV. The samples of antibacterial adhesion were also divided into three groups: PMMA‐n, PMMA‐v and dual carrier‐v. The samples of the PMMA‐v and dual carrier‐v were immersed in sterile polyethylene containers containing 10 mL SBF (pH 7.4) at 37 °C, respectively. The SBF solution was semiquantitatively refreshed every 24 h after the samples immersed in SBF. The drug delivery samples were removed from the SBF after 0, 7 and 14 days of immersion in the SBF. In the evaluation of antibacterial adhesion, the PMMA‐unloaded vancomycin was used as a blank control. Then the three kinds of samples were immersed in 10 mL of the bacterial suspension adjusted to 3 × 10^8^ CFUs·mL^−1^ for 24 h at 37° C, respectively. After immersion for 24 h, the samples were obtained and debrided twice with PBS to remove bacteria that did not adhere to the surfaces of the samples. Then the samples were immobilized in a 2.5% aqueous solution of glutaraldehyde for 24 h and dehydrated for 10 min with different gradients of ethanol solution (30%, 50%, 70%, 90% and 100%). After the sequential dehydration steps, the samples were dried overnight at room temperature. The bacteria adhesion on the sample surface was observed by SEM. For each sample, imaging was carried out through SEM at five different random locations. The average number of adherent bacteria at the above five locations was obtained for evaluation of antibacterial adhesion.

### Statistical analysis

All data were reported as means ± SD, unless otherwise stated. Student’s *t*‐test was performed between two groups, and one‐way analysis of variance (ANOVA) with Tukey’s *post hoc* test was performed among multiple groups. Statistical significance was built with a *P*‐value <0.05.

## Results

### The antibiotic release profiles *in vitro*

**Fig. 2:**
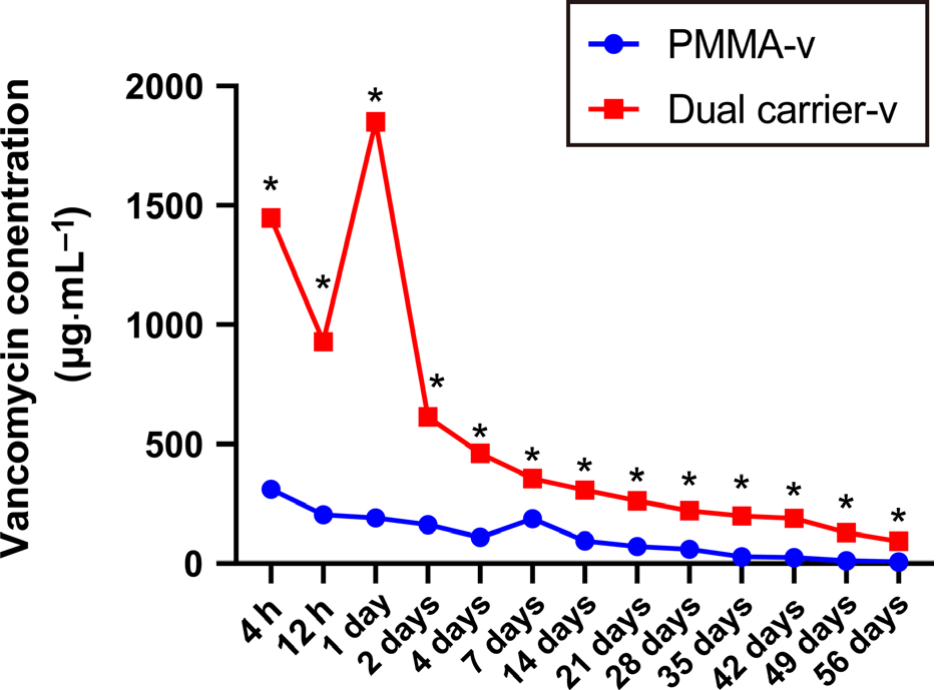
Characterization of vancomycin release kinetics from the dual carrier‐v and PMMA‐v. *N* = 5; mean ± SD; Student’s *t*‐test, *P < 0.05.

Figure 2 shows the vancomycin release profiles of the dual carrier‐v and PMMA‐v. Vancomycin was gradually released from dual carrier‐v and PMMA‐v up to about 8 and 6 weeks, respectively. During the releasing time, the concentration of vancomycin released from the dual carrier‐v was higher than the concentration of vancomycin released by PMMA‐v (*P* < 0.05). In addition, there was a recoil releasing of vancomycin at day 1 from dual carrier‐v.

### Antibacterial properties evaluated by inhibition zone

**Fig. 3:**
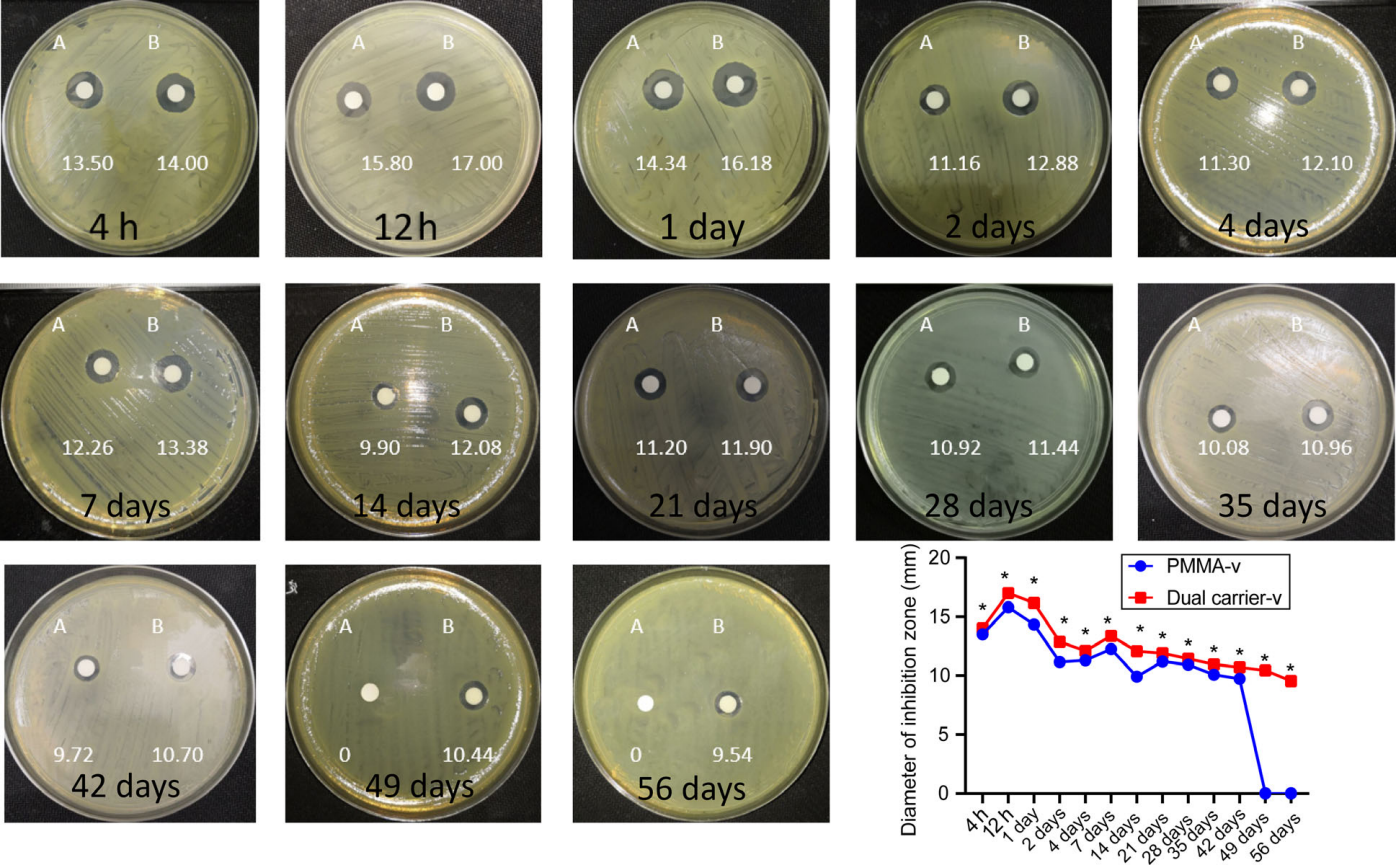
Representative images and statistics of inhibition zones of *S. aureus* (mm) at different time points. (A) PMMA‐v. (B) Dual carrier‐v. *n* = 5; mean ± SD; Student’s *t*‐test, *P < 0.05.

Figure 3 shows the inhibition zone of vancomycin at different elution time points against bacteria on M‐H agar plate. The inhibition zones were measured surrounding the filter paper discs impregnated with serial vancomycin SBF eluents at different time points. In each assay at different elution time points, the inhibition zone of the dual carrier‐v was larger than the inhibition zone of the PMMA‐v (*P <* 0.05). In this experiment, no growth inhibition zone was observed from the PMMA‐n.

### Antibacterial properties evaluated by colony inhibition

**Fig. 4:**
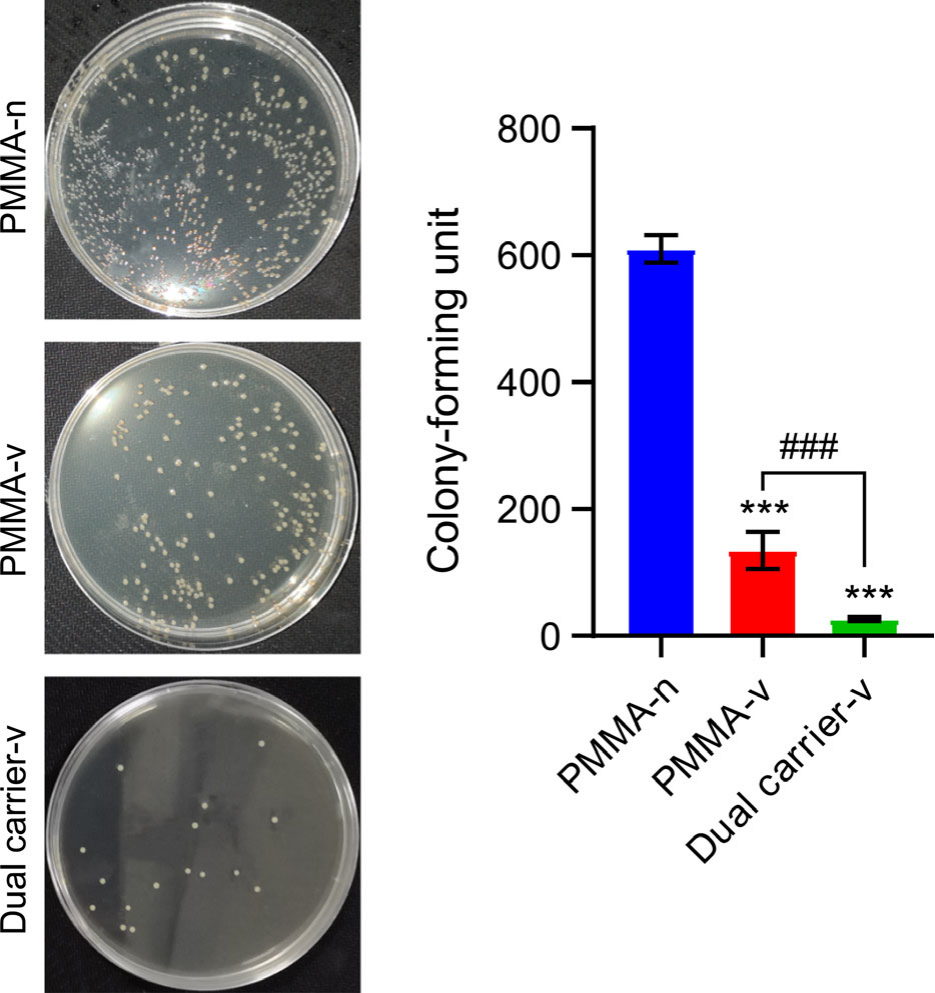
Representative images and statistics of CFUs of* S. aureus*. *n* = 5; mean ± SD; one‐way ANOVA with Tukey’s *post hoc* test, ***P < 0.001, ^###^ *P* < 0.001.

Figure 4 shows the colony inhibition of vancomycin elution at different time points against bacteria on the M‐H agar plate. The average CFUs of PMMA‐n, PMMA‐v and dual carrier‐v were 610, 135 and 27, respectively (*P* < 0.001). The inhibition rate of the dual carrier‐v was 95.57%, whereas that of the PMMA‐v was 77.87%.

### Antibacterial adhesion evaluated by SEM

**Fig. 5:**
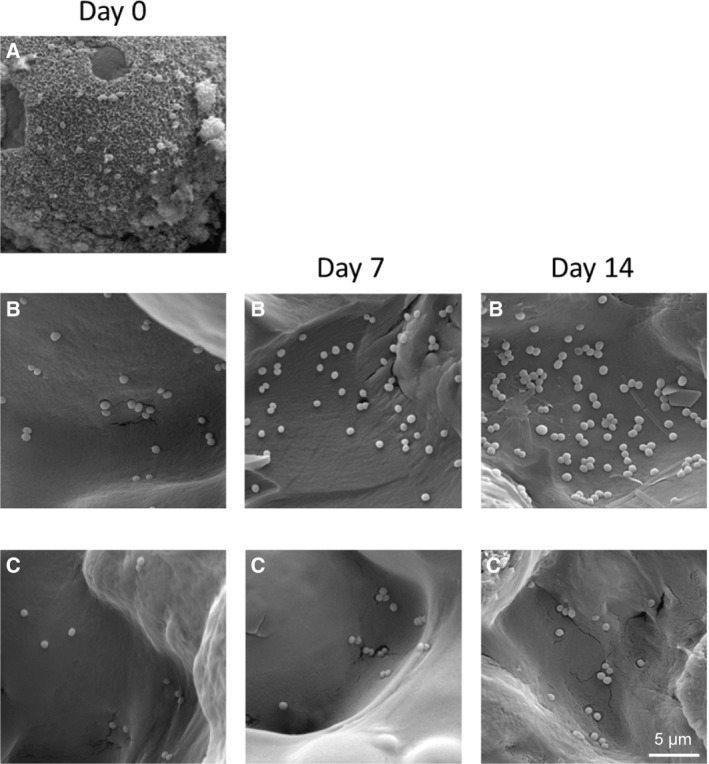
Scanning electron micrograph of antibacterial adhesion at the indicated times after application. (A) PMMA‐n. (B) PMMA‐v. (C) Dual carrier‐v. Scale bar: 5 μm.

To better observe the antibacterial adhesion of the PMMA‐n, PMMA‐v and dual carrier‐v at different time points, we applied SEM. Figure 5 shows that biofilm had formed on the PMMA‐n surface, whereas no biofilm formed on the PMMA‐v and dual carrier‐v surfaces. Due to the biofilm formation, the exact amount of bacterial adhesion could not be obtained on the surface of the PMMA‐n. The PMMA‐v without immersion in SBF had detectable bacterial adhesion, and the amount of bacterial adhesion was observed to increase with increasing immersion time from 28 to 110 cells (*P* < 0.05), whereas dual carrier‐v did not show a significant increase of bacterial adhesion with increasing immersion time within 14 days (*P > *0.05; Table 1). More importantly, the dual carrier‐v was shown to be more antibacterial effective at each time point after 0, 7 and 14 days of immersion into the SBF compared with PMMA‐v (*P* < 0.05; Table 1).

**Table 1:** Adherent bacteria on the surface of PMMA‐v and dual carrier‐v (*n* = 5).

Groups	CFUs of adherent bacteria		
	Day 0	Day 7	Day 14
Dual carrier‐v	12 ± 5.43*	18 ± 5.86*	20 ± 5.68*
PMMA‐v	28 ± 8.82**^,^***	46 ± 7.16**^,^***	110 ± 7.48**^,^***

* *P* > 0.05 (one‐way ANOVA with Tukey’s *post hoc* test) among the different time points in the dual carrier‐v group

** *P* < 0.05 (one‐way ANOVA with Tukey’s *post hoc* test) among the different time points in the PMMA‐v group

*** *P* < 0.05 (Student’s *t*‐test) between groups in the same time point.

## Discussion

The elution and antibacterial effects of massive properties of PMMA, calcium sulfate or other local antibiotic loadings were well studied. However, few studies have reported antibiotic release properties and antibacterial properties of composite drug delivery system consisting of dual carrier‐v. In our previous clinical study, the dual carrier‐v could gain more effectively clinical control of infection for osteomyelitis [3]. However, we did not know the antibiotic release and antibacterial effects of dual carrier‐v. Thus, this study aimed to explore the antibiotic release characteristics and the antibacterial properties of the dual carrier‐v, with PMMA‐v as control. In this study, the dual carrier‐v showed several merits, and it can play a synergistic antibacterial effect as compared with PMMA‐v.

Compared with PMMA‐v, the vancomycin elution of dual carrier‐v was much higher and gained long‐lasting effective antibiotic concentration during the study, especially in the early stages of drug release experiments. Antibiotic‐loaded PMMA was most commonly used for chronic osteomyelitis management because of its localized antibiotic‐releasing effects, and has been traditionally used for preventative and treatment strategies for orthopedic‐related infections. Different from the parenteral/oral administration of antibiotics, the PMMA can be easily loaded antibiotics from the bulk to the surface at the site of infection [15]. However, the major problem of PMMA is the antibiotic resistance caused by the transitory peak releasing at the very beginning [16], and only up to 10% of drugs will be eluted [17]. Because of these shortcomings from antibiotic‐loaded PMMA, alternatives to PMMA for more sustained and higher concentrations of antibiotic delivery are suggested. A calcium sulfate‐based cargo carrier has been launched for its long‐lasting release and biocapacity over PMMA. More importantly, the low gradual drug release rate of calcium sulfate helps prevent the occurrence of antibiotic resistance. In this study, the concentration of vancomycin elution from the dual carrier‐v was much higher than that from PMMA‐v. This may be related to the absorbable properties and gradual degradation characteristics of calcium sulfate. In addition, absorbable calcium sulfate is preferred to PMMA for the treatment of dead space infection, especially elution kinetics of antibiotics [18, 19].

Our results of inhibition zone and colony inhibition recognized that the dual carrier‐v was optimal to inhibit the *S. aureus* growth with significant statistical difference as compared with PMMA‐v (*P* < 0.05). Due to the absolute degradation and complete elution of the calcium sulfate cubes, greater inhibition zone was achieved in the dual carrier‐v than PMMA‐v. As a standard treatment of chronic osteomyelitis, PMMA‐v showed some drawbacks because of its release kinetics, in particular to the abruptly eluted antibiotics, then declined gradually [8]. Substantially, biofilm formation will occur because of the colonization formed by the bacteria, which antibiotics normally find hard to penetrate [20], and thus the chronic infection will recover. However, dual carrier‐v can overcome this deficiency. The long‐term releasing effects of dual carrier‐v can be achieved and maintained through its structure. In each assay at different time points, the inhibition zone of the dual carrier‐v was larger than the inhibition zone of the PMMA‐v (*P* < 0.05). It may be related to the effective release of antibiotics for a longer period in the former.

Due to the protection against antibiotic penetration of bacterial biofilm for bacteria, managing chronic osteomyelitis is still a big challenge [21, 22]. The biofilm formation is a continuous process. It involves several distinct stages, including initial adhesion, adsorption and proliferation between cells, biofilm maturation and bacterial dispersal processes [23, 24]. The bacteria must first adhere to the surface of the solid to form a mature biofilm. Therefore, attachment from free single bacteria to the surface of the attachment is a critical stage in biofilm formation [25]. In this study, biofilm was detectable on the PMMA‐n and was not observed on either PMMA‐v or the dual carrier‐v. Crucially, as well as eliminating or preventing bacterial biofilm formation, the dual carrier‐v demonstrated a significant reduction in bacterial colonization on its surface as compared with PMMA‐v. The amount of bacterial adhesion and colonization on the PMMA‐v surface was significantly aggrandized with increasing immersion time within 14 days. However, it did not significantly increase on the surface of the dual carrier‐v. This result may be related to the effective increase and an extension of the release of vancomycin from the dual carrier‐v, which was accomplished through implanting calcium sulfate cubes containing vancomycin into the PMMA spacer. Bacteria can easily attach and adhere on the plain PMMA when there are insufficient antibiotics [26], which results from the insufficient elution kinetics rather than other materials [27]. In contrast, adequate concentrations of antibiotics are key factors because of the sublethal concentration effects [29]. Thus, researchers have improved the ingredients of PMMA to make better pharmacokinetics [29, 30]. In this work, compared with the PMMA‐v, the dual carrier‐v can optimize the vancomycin release, especially in the early stages of drug release experiments. Calcium sulfate‐based antibiotic carriers have presented its antibiofilm formation because of its long‐lasting antibacterial capacity [31]. More importantly, the dual carrier‐v can yield synergistic roles. Because of its inherently nonbiodegradable properties, PMMA itself not only lasts the time of degradation but also supports the bone structure during osteomyelitis management [32, 33]. Antibiotic‐loaded calcium sulfate can offer an effective concentration of antibiotics in the infection during degradation [34, 35]. However, calcium sulfate is not a structural supporter because the rapid occurrence of calcium sulfate absorption *ex vitro* would impair the mechanical strength [36, 37]; therefore, a deep infection would be a substantial harmful consequence [38].

## Conclusions

In this study, the dual poly(methyl acrylate, methyl methacrylate) (PMMA)/calcium sulfate carrier of vancomycin (dual carrier‐v) is more effective than PMMA‐vancomycin (PMMA‐v) at inhibiting Staphylococcus aureus growth *in vitro*. Further research on the usage of this material may be necessary for orthopedic infection.

## Conflict of interest

The authors declare no conflict of interest.

## Author contributions

Shanchao L, CZ and Shixing L conceived and designed the project. Shancao L, TJ, Long L, YY and XY acquired the data. Luo L, JL and ZC analyzed and interpreted the data. Shanchao L and TJ wrote the paper.
